# *In crystallo* activity tests with latent apple tyrosinase and two mutants reveal the importance of the mutated sites for polyphenol oxidase activity

**DOI:** 10.1107/S2053230X17010822

**Published:** 2017-07-28

**Authors:** Ioannis Kampatsikas, Aleksandar Bijelic, Matthias Pretzler, Annette Rompel

**Affiliations:** a Universität Wien, Fakultät für Chemie, Institut für Biophysikalische Chemie, Althanstrasse 14, 1090 Wien, Austria

**Keywords:** polyphenol oxidase, plant tyrosinase, latent pro-enzyme, type 3 copper enzyme, activity controllers

## Abstract

Tyrosinases are type 3 copper enzymes that belong to the polyphenol oxidase (PPO) family and are able to catalyze both the *ortho*-hydroxylation of monophenols and their subsequent oxidation to *o*-quinones, which are precursors for the biosynthesis of colouring substances such as melanin. The first plant pro-tyrosinase from *Malus domestica* (*Md*PPO1) was recombinantly expressed in its latent form (56.4 kDa) and mutated at four positions around the catalytic pocket which are believed to influence the activity of the enzyme. Mutating the amino acids, which are known as activity controllers, yielded the mutants *Md*PPO1-Ala239Thr and *Md*PPO1-Leu243Arg, whereas mutation of the so-called water-keeper and gatekeeper residues resulted in the mutants *Md*PPO1-Glu234Ala and *Md*PPO1-Phe259Ala, respectively. The wild-type enzyme and two of the mutants, *Md*PPO1-Ala239Thr and *Md*PPO1-Phe259Ala, were successfully crystallized, leading to single crystals that diffracted to 1.35, 1.55 and 1.70 Å resolution, respectively. All crystals belonged to space group *P*2_1_2_1_2_1_, exhibiting similar unit-cell parameters: *a* = 50.70, *b* = 80.15, *c* = 115.96 Å for the wild type, *a* = 50.58, *b* = 79.90, *c* = 115.76 Å for *Md*PPO1-Ala239Thr and *a* = 50.53, *b* = 79.76, *c* = 116.07 Å for *Md*PPO1-Phe259Ala. *In crystallo* activity tests with the crystals of the wild type and the two mutants were performed by adding the monophenolic substrate tyramine and the diphenolic substrate dopamine to crystal-containing drops. The effects of the mutation on the activity of the enzyme were observed by colour changes of the crystals owing to the conversion of the substrates to dark chromophore products.

## Abstract 2

*M. domestica* polyphenol oxidase 1 (*Md*PPO1) was recombinantly expressed in its latent form (Lys1–Ser504) and successfully mutated at four different positions around the active centre which have been proposed to be decisive for the activity of the enzyme. The wild-type *Md*PPO1 and two of the mutants were successfully crystallized. *In crystallo* activity tests demonstrated the importance of these amino acids for the activity of the enzyme.

## 1. Introduction   

Tyrosinases (TYRs) are members of the type 3 copper enzyme family, which also includes catechol oxidases (COs) and aurone synthase (AUS). TYRs, COs and AUS are summarized under the umbrella term polyphenol oxidases (PPOs). These enzymes are widely distributed in bacteria, fungi, plants and animals (Mayer, 2006 ▸; Tran *et al.*, 2012 ▸; Kaintz, Mauracher *et al.*, 2014 ▸; Pretzler *et al.*, 2015 ▸). Tyrosinases are able to catalyze the *ortho*-hydroxylation of monophenols to *o*-diphenols (monophenolase activity; EC 1.14.18.1) coupled with the subsequent two-electron oxidation of *o*-diphenols to the corresponding *o*-quinones (diphenolase activity; EC 1.10.3.1) (Mason, 1955 ▸; Mayer *et al.*, 1966 ▸). The *o*-diphenols formed in the hydroxylation step remain in the active centre and are oxidized to the quinonic state (Ramsden & Riley, 2014 ▸). During the TYR-mediated hydroxylation and oxidation of one molecule of monophenol, one molecule of dioxygen is reduced to water. COs lack the monophenolase activity and are thus only capable of oxidizing *o*-diphenols. The resulting *o*-quinones, which are the products of all PPOs, are highly reactive and represent the starting material for the biosynthesis of colouring substances such as melanin (Mason, 1948 ▸; Rodríguez-López *et al.*, 1992 ▸). Thus, both TYRs and COs are involved in the browning reaction of fruits and vegetables, which represents a major concern in the food industry. The third type 3 copper enzyme AUS from the petals of *Coreopsis grandiflora* (*cg*AUS1; Molitor, Mauracher, Pargan *et al.*, 2015 ▸; Kaintz, Molitor *et al.*, 2014 ▸; Kaintz *et al.*, 2015 ▸) shows monophenolase activity towards chalcone substrates (for example isoliquiritigenin) but not towards classical tyrosinase substrates such as tyrosine and tyramine, and has therefore been classified as a CO. Moreover, *cg*AUS1 has been crystallized and structurally analyzed (Molitor, Mauracher & Rompel, 2015 ▸; Molitor, Mauracher & Rompel, 2016 ▸; Molitor, Bijelic & Rompel, 2016 ▸).

**Figure 1:**
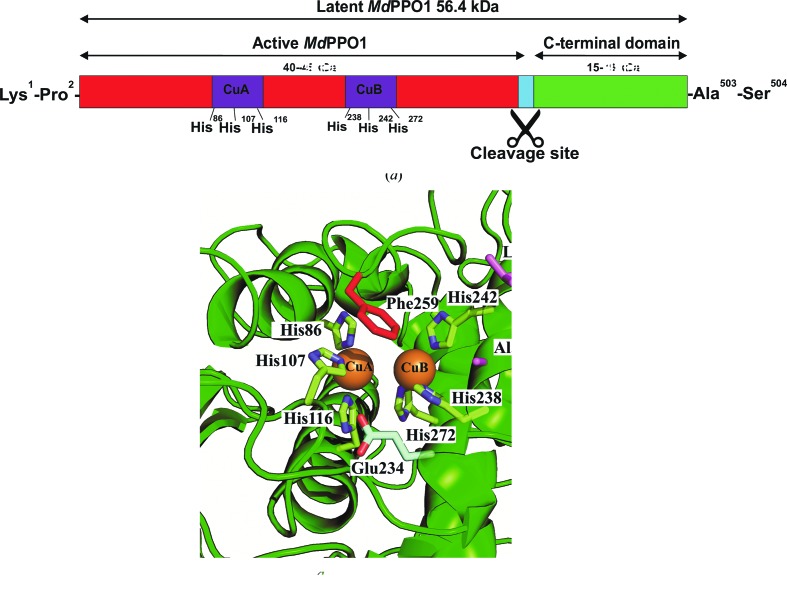
(*a*) Schematic representation of the primary structure of recombinant latent *Md*PPO1 (56.4 kDa). The active domain of the enzyme is coloured red and the conserved regions (histidines) of the copper-coordinating motifs are presented in purple. The C-terminal domain is coloured green and the putative position of proteolytic activation between the active domain and the C-terminus is displayed in blue. (*b*) Structural representation of the active centre of the wild-type *Md*PPO1 from homology modelling shows the conserved histidines coordinating CuA (His86, His107 and His116) and CuB (His238, His242 and His272) and the positions of the mutated residues, namely the two activity controllers alanine (Ala239) and leucine (Leu243), both in purple, the water keeper glutamic acid (Glu234) in light green and the gatekeeper phenylalanine (Phe259) in red. The homology model of *Md*PPO1 was obtained in the same way as described in Kampatsikas *et al.* (2017 ▸).

Plant PPOs are known to be expressed as latent pro-enzymes *in vivo* exhibiting a molecular weight of ∼64–68 kDa and consist of three domains: a signal peptide (in a minority of plant PPOs) or a transit peptide containing a thylakoid transfer domain (in the majority of plant PPOs) (∼4–9 kDa), a catalytically active domain (∼40 kDa) and a shielding C-terminal domain (∼15–19 kDa) (Tran *et al.*, 2012 ▸; Fig. 1 ▸ *a*). The enzyme is activated *in vivo* by a hitherto unknown proteolytic cleavage process (Fig. 1 ▸ *a*) in which the C-terminus is separated from the active domain, leading to a substrate-accessible catalytic pocket (Flurkey & Inlow, 2008 ▸). The enzyme can also be activated *in vitro* using proteases (Gandía-Herrero *et al.*, 2005*b* ▸; Pretzler *et al.*, 2017 ▸; Kampatsikas *et al.*, 2017 ▸), an acidic pH (Valero & García-Carmona, 1992 ▸; Kampatsikas *et al.*, 2017 ▸), fatty acids (Sugumaran & Nellaiappan, 1991 ▸) or detergents such as sodium dodecyl sulfate (SDS; Gandía-Herrero *et al.*, 2005*a* ▸; Kampatsikas *et al.*, 2017 ▸). The maturation process activating the enzyme *in vivo* and the function of the C-terminal domain are still a matter of debate.

Another ongoing discussion is the structural basis for the lack of monophenolase activity in COs, as the crystal structures of diverse COs (Klabunde *et al.*, 1998 ▸; Virador *et al.*, 2010 ▸; Molitor, Mauracher & Rompel, 2015 ▸, 2016 ▸) and TYRs (Matoba *et al.*, 2006 ▸; Sendovski *et al.*, 2011 ▸; Ismaya, Rozeboom, Schurink *et al.*, 2011 ▸; Ismaya, Rozeboom, Weijn *et al.*, 2011 ▸; Mauracher *et al.*, 2014*a* ▸,*b* ▸; Zekiri, Bijelic *et al.*, 2014 ▸; Bijelic *et al.*, 2015*a*, ▸ *b* ▸) revealed an astoundingly high similarity of their active sites. In recent decades some theories based on the position and/or presence of certain amino acid residues within the active site have been postulated, but none of these were able to identify structural features which could explain the lack of monophenolase activity in COs (Kanteev *et al.*, 2015 ▸; Pretzler & Rompel, 2017 ▸). One of these amino acids is the so-called gatekeeper residue, which is located directly above the first copper in the active site (CuA) and acts as a stabilizer for incoming substrates *via* hydrophobic T-shaped π–π inter­actions (Bijelic *et al.*, 2015*a*, ▸ *b* ▸; Molitor, Mauracher & Rompel, 2016 ▸). Another residue is the water keeper, which is assumed to stabilize a conserved water molecule that is responsible for the putatively monophenolase-decisive deprotonation of incoming monophenolic substrates (Goldfeder *et al.*, 2014 ▸).

Recently, cDNA encoding a PPO pro-enzyme has been cloned from apple leaves (*Malus domestica*; *Md*PPO1; ENA LT718522) and was heterologously expressed in *Escherichia coli* (Kampatsikas *et al.*, 2017 ▸). *Md*PPO1 was subsequently purified and characterized, revealing monophenolase activity towards tyramine and tyrosine and thus classifying *Md*PPO1 as a TYR (Kampatsikas *et al.*, 2017 ▸). The study also revealed the presence of further amino acids that are relevant for the enzyme’s activity, which are located next to the first and second histidines of copper B (CuB) and are therefore termed activity controllers. Together with the two abovementioned residues, they can control the activity of the enzyme (monophenolase or diphenolase activity).

In this study, four mutants of *Md*PPO1 were produced recombinantly and purified in order to study their effect on the activity of the enzyme, namely *Md*PPO1-Ala239Thr, *Md*PPO1-Leu243Arg, *Md*PPO1-Glu234Ala and *Md*PPO1-Phe259Ala. The first two mutations, *Md*PPO1-Ala239Thr and *Md*PPO1-Leu243Arg, affect the nonconserved activity-controller residues Ala239 and Leu243 (Kampatsikas *et al.*, 2017 ▸). *Md*PPO1-Glu234Ala is the result of mutating the water keeper Glu234, and mutation of the gatekeeper Phe259 yielded the mutant *Md*PPO1-Phe259Ala (see Fig. 1 ▸ *b*). During the subsequent crystallization trials, the wild-type *Md*PPO1 and two of the four mutants, *Md*PPO1-Ala239Thr and *Md*PPO1-Phe259Ala, were successfully crystallized, representing the first crystals of recombinantly expressed latent plant TYR. Crystals of wild-type *Md*PPO1, *Md*PPO1-Ala239Thr and *Md*PPO1-Phe259Ala diffracted to 1.35, 1.55 and 1.70 Å resolution, respectively. Furthermore, the crystals were soaked with a monophenolic (tyramine) and a diphenolic (dopamine) substrate using sodium dodecyl sulfate (SDS) as an activator in order to perform *in crystallo* activity tests. This is possible as the products of the PPO reaction, quinones, further react to dark chromophores, leading to the browning of the crystals. This procedure was well tolerated by the crystals without damage. The *in crystallo* activity tests revealed the importance of the mutated positions, as both mutants significantly affect the activity of the enzyme, providing valuable insights into the catalytic mechanism of plant PPOs.

## 2. Materials and methods   

### 2.1. Preparation of *Md*PPO1   

**Figure 2:**
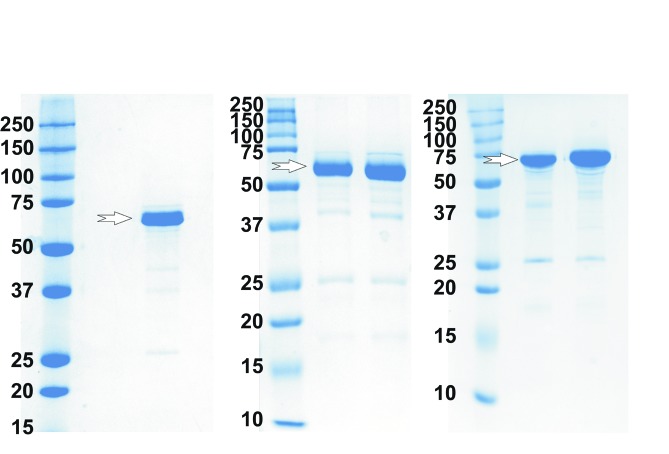
SDS–PAGE with 7 µg of enzyme under reducing conditions. (*a*) Wild-type *Md*PPO1, (*b*) *Md*PPO1-Ala239Thr and *Md*PPO1-Leu243Arg, and (*c*) *Md*PPO1-Glu234Ala and *Md*PPO1-Phe259Ala. The arrows indicate the position of the enzyme. Lanes *M* contain molecular-mass markers (labelled in kDa).

Production of the target enzyme *Md*PPO1 was performed as described previously (Kampatsikas *et al.*, 2017 ▸). Briefly, the gene encoding latent *Md*PPO1 (without the signal peptide; residues Lys1–Ser504) was inserted into the expression vector pGEX-6P-1 (GE Healthcare) and N-terminally fused with the glutathione *S*-transferase (GST) tag of the vector. A protease (HRV3C) recognition sequence (LEVLFQ|GP) was present between the GST tag and the target enzyme for subsequent tag removal. Competent *E. coli* BL21 cells were transformed with the vector and cultured at 310 K until an OD_600_ of 0.6–0.8 was reached, at which point the temperature was reduced to 293 K and protein expression was induced by the addition of 0.5 m*M* isopropyl β-d-1-thiogalactopyranoside (IPTG) and 0.5 m*M* copper(II) sulfate. Expression was continued for additional 30–40 h at 293 K until the cell density reached an OD_600_ of 7–10. The cells were collected by centrifugation and lysed by the freeze–thaw technique. The enzyme was purified using a GSTrap FF column (GE Healthcare) followed by GST-tag cleavage with HRV3C protease. Further purification was performed using a second GSTrap FF column. The protein was concentrated to about 5–10 mg ml^−1^ in a buffer consisting of 50 m*M* Tris–HCl, 200 m*M* NaCl pH 7.5. Target-enzyme production is summarized in Table 1 ▸. For the production of the four mutants, primers were designed which are summarized in Table 1 ▸. The clone of the wild type in the pGEX-6P-1 vector was used as a template for the construction of the mutants and the Q5 Site-Directed Mutagenesis Kit (NEB) was used to prepare the mutants. Expression and purification of the mutants was performed as described for the wild-type *Md*PPO1; the purity level of the produced mutants was controlled with SDS–PAGE (Fig. 2 ▸).

### 2.2. Protein crystallization   

**Figure 3:**
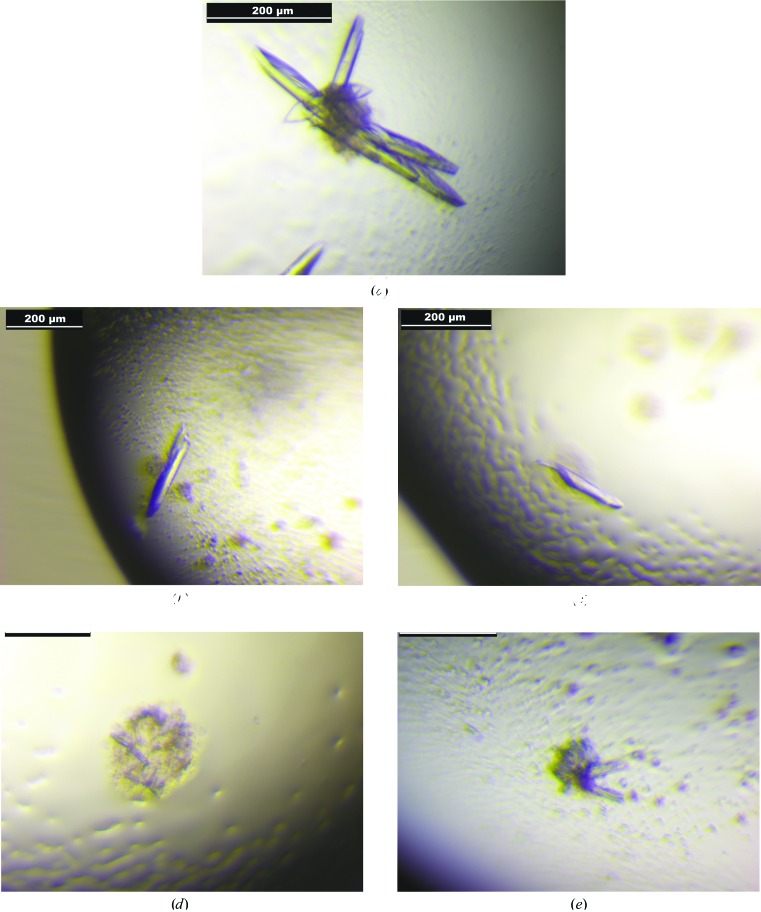
Crystals of (*a*) wild-type *Md*PPO1, (*b*) *Md*PPO1-Ala239Thr, (*c*) *Md*PPO1-Phe259Ala, (*d*) *Md*PPO1-Glu234Ala and (*e*) *Md*PPO1-Leu243Ala.

Initial screening for suitable crystallization conditions for wild-type *Md*PPO1 was performed by the sitting-drop vapour-diffusion method using a nanodispenser robot (Gryphon, Art Robbins) and 96-well Crystal Quick plates (Greiner Bio-One). A wide range of crystallization conditions were screened by using a variety of commercially available screening kits (JBScreen Classic 1–10, JBScreen Membrane 1–3 and Pi-PEG Screen HTS from Jena Bioscience). The screening procedure yielded some promising hits, which were further optimized manually by applying the hanging-drop vapour-diffusion technique using 15-well EasyXtal plates (Qiagen). Single crystals of wild-type *Md*PPO1 and of the mutants *Md*PPO1-Ala239Thr and *Md*PPO1-Phe259Ala (Fig. 3 ▸) were grown at 20°C by mixing 1 µl protein solution (5–10 mg ml^−1^) with 2 µl reservoir solution (50 m*M* Tris–HCl pH 7.0, 19–21% PEG 3350). Crystals usually appeared after 10–15 d. Crystallization information is summarized in Table 2 ▸. Crystallization trials of the mutants *Md*PPO1-Leu243Arg and *Md*PPO1-Glu234Ala applying the above-described methods yielded only low-quality crystals which were not suitable for adequate data collection (Fig. 3 ▸).

### 2.3. Data collection and processing   

Crystals of latent wild-type *Md*PPO1, *Md*PPO1-Ala239Thr and *Md*PPO1-Phe259Ala were mounted in nylon loops and flash-cooled in liquid nitrogen after quick soaking in cryoprotectant solution composed of 50 m*M* Tris–HCl pH 7.5, 200 m*M* NaCl, 20% PEG 3350, 20–25% PEG 1500. Data collection for wild-type *Md*PPO1 was carried out at 100 K on beamline ID23 at the ESRF, Grenoble, France (Table 3 ▸), whereas data for the mutants *Md*PPO1-Ala239Thr and *Md*PPO1-Phe259Ala were collected at 100 K on beamline ID30A-3/MASSIF-3 at the ESRF, Grenoble, France (Table 3 ▸). The data sets were processed (indexing, integration and scaling) with *XDS* (Kabsch, 2010 ▸). All crystals belonged to space group *P*2_1_2_1_2_1_, which was determined using *POINTLESS* (Evans, 2011 ▸) from the *CCP*4 suite (Winn *et al.*, 2011 ▸).

### 2.4. *In crystallo* activity tests   

**Figure 4:**
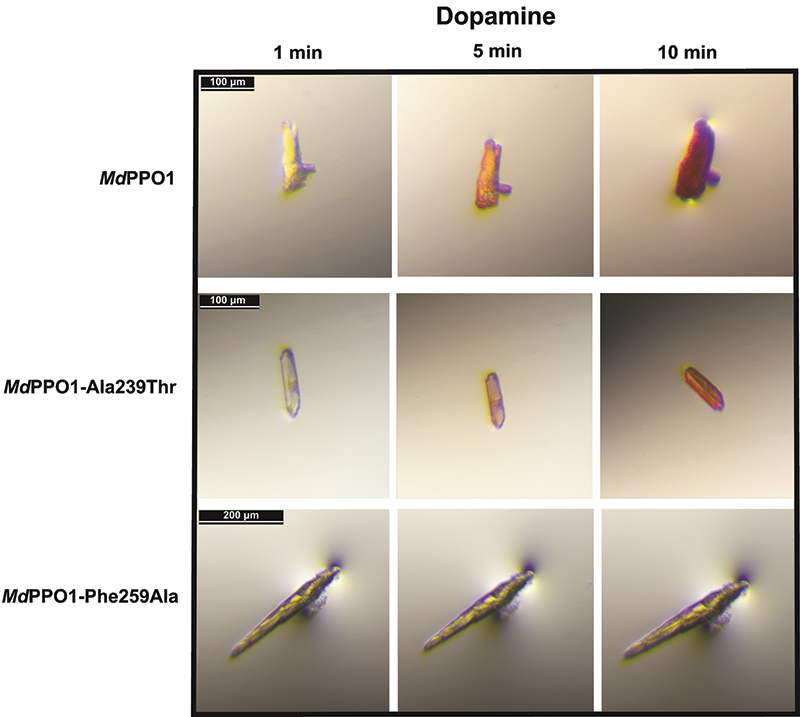
In *crystallo* activity tests: photographs of crystals of wild-type *Md*PPO1 and the mutants *Md*PPO1-Ala239Thr and *Md*PPO1-Phe259Ala with 10 m*M* dopamine and 3 m*M* SDS as an activator. Crystal photographs were taken after 1, 5 and 10 min, respectively.

**Figure 5:**
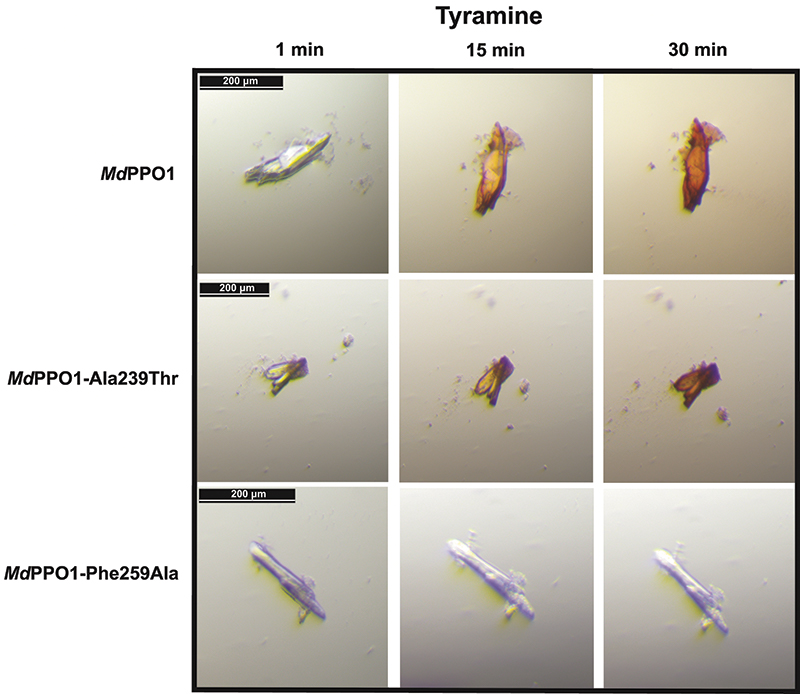
In *crystallo* activity tests: photographs of crystals of wild-type *Md*PPO1 and the mutants *Md*PPO1-Ala239Thr and *Md*PPO1-Phe259Ala with 10 m*M* tyramine and 3 m*M* SDS as an activator. Crystal photographs were taken after 1, 15 and 30 min, respectively.

PPO reactions produce unstable quinones which further polymerize to chromophoric substances, and therefore it was possible to detect differences in activity between wild-type *Md*PPO1 and the mutants *in crystallo*. For this reason, crystals of the wild type and its mutants were transferred to drops consisting of the mother liquor with an additional 3 m*M* SDS, which is needed to activate the latent enzyme, and 10 m*M* substrate, namely tyramine or dopamine, to detect monophenolase and diphenolase activity, respectively. After the transfer of a crystal to a substrate-containing drop, pictures were taken during the reaction with dopamine (Fig. 4 ▸) or tyramine (Fig. 5 ▸). In this way, the conversion of the crystals was followed and differences were observed between the wild type and the mutants in the intensity and speed of the colouration.

## 3. Results and discussion   

The method described by Kampatsikas *et al.* (2017 ▸) was used to produce sufficient amounts (225 mg per litre of culture) of wild-type *Md*PPO1 in its latent form as well as the mutants *Md*PPO1-Ala239Thr, *Md*PPO1-Leu243Arg, *Md*PPO1-Glu234Ala and *Md*PPO1-Phe259Ala. For crystallization reasons, wild-type *Md*PPO1 was screened with a total of 408 crystallization conditions, which led to four promising hits: (i) 4.3%(*w*/*v*) PEG 4000 and 25%(*w*/*v*) PEG 1500 in 50 m*M* sodium acetate buffer pH 4.8; (ii) 5.7%(*v*/*v*) PEG 350 monomethyl ether (MME) and 21.4%(*w*/*v*) PEG 3000 in 50 m*M* MES buffer pH 6.0; (iii) 2.5%(*w*/*v*) PEG 1000 and 25.7%(*w*/*v*) PEG 2000 MME in 50 m*M* sodium acetate buffer pH 4.8; and (iv) 2.5%(*w*/*v*) PEG 1500 and 25.7%(*w*/*v*) PEG 3000 in 50 m*M* sodium acetate buffer pH 5.2. The initial crystals were further optimized, resulting in large but twinned crystals. However, it was possible to separate single crystals from the twinned crystal clusters, thus obtaining single crystals that were suitable for data collection (Fig. 3 ▸ *a*). Similar conditions were tested for the crystallization of the four mutants. Crystals of *Md*PPO1-Ala239Thr and *Md*PPO1-Phe259Ala were obtained under the same conditions as used for the wild type (Table 2 ▸, Figs. 3 ▸ *b* and 3 ▸ *c*), while *Md*PPO1-Glu234Ala and *Md*PPO1-Leu243Ala only formed microcrystals that hardly diffracted and failed to produce evaluable data during the X-ray diffraction experiment (Figs. 3 ▸ *d* and 3 ▸ *e*). The crystals were obtained from solutions containing PEGs of different molecular masses as the precipitation agent. The utilization of PEG as a precipitation agent is the only constant among all published crystallization conditions of fungal tyrosinases (Ismaya, Rozeboom, Schurink *et al.*, 2011 ▸; Ismaya, Rozeboom, Weijn *et al.*, 2011 ▸; Mauracher *et al.*, 2014*a* ▸,*b* ▸) and bacterial tyrosinases (Matoba *et al.*, 2006 ▸; Sendovski *et al.*, 2011 ▸) as well as of plant catechol oxidases (Klabunde *et al.*, 1998 ▸; Virador *et al.*, 2010 ▸; Molitor, Mauracher & Rompel, 2015 ▸) and plant tyrosinases (Zekiri, Molitor *et al.*, 2014 ▸; Zekiri, Bijelic *et al.*, 2014 ▸; Bijelic *et al.*, 2015*a*, ▸ *b* ▸). The obtained crystals of wild-type *Md*PPO1 and the mutants *Md*PPO1-Ala239Thr and *Md*PPO1-Phe259Ala diffracted X-rays to 1.35, 1.55 and 1.70 Å resolution, respectively. Data-collection and processing statistics are summarized in Table 3 ▸, revealing that the data sets are of good quality. Phasing applying the molecular-replacement approach and subsequent structure determination is currently under way. Several promising models for the molecular-replacement step are available according to the results of a *BLAST* search (Altschul *et al.*, 1990 ▸): tyrosinase from *Juglans regia* (sequence identity 66.6%; UniProt C0LU17; PBD entry 5ce9; Zekiri, Bijelic *et al.*, 2014 ▸; Bijelic *et al.*, 2015*a*, ▸ *b* ▸), catechol oxidase from *Vitis vinifera* (sequence identity 59.2%; UniProt P43311; PDB entry 2p3x; Virador *et al.*, 2010 ▸), catechol oxidase from *Ipomoea batatas* (sequence identity 53.0%; UniProt Q9ZP19; PDB entry 1bt3; Klabunde *et al.*, 1998 ▸) and aurone synthase from *C. grandiflora* (sequence identity 43.0%; UniProt A0A075DN54; PDB entry 4z11; Molitor, Mauracher & Rompel, 2015 ▸, 2016 ▸). The latter has previously been used for homology modelling of *Md*PPO1 in Kampatsikas *et al.* (2017 ▸).

The *in crystallo* activity test showed that the wild type is active towards both substrates, as expected and described in Kampatsikas *et al.* (2017 ▸). This was obvious owing to the rapid and intense colouration of the wild-type crystal with both dopamine (Fig. 4 ▸) and tyramine (Fig. 5 ▸). *Md*PPO1-Ala239Thr represents a mutation that affects one of the previously described activity-controller residues, namely Ala239, which was mutated from a small hydrophobic alanine to a polar threonine residue in order to influence the activity, as it was suggested that the polarity of the environment of the active site might be an important factor in the activity specificity of plant PPOs (Fig. 1 ▸ *b*). According to the *in crystallo* activity test, this mutation has only a minor impact on the diphenolase activity as the speed and intensity of crystal browning is comparable to that of the wild type (Fig. 4 ▸). However, this mutation led to significantly slower (1–2 min) and less intense browning with the monophenol tyramine, indicating a decrease in monophenolase activity. The crystal of the mutant *Md*PPO1-Ala239Thr maintains the monophenolase activity, indicating that this mutation is not sufficient to adequately diminish the reaction with tyramine. The mutation of the bulky gatekeeper Phe259 to a small alanine (Phe259Ala) led to a dramatic change in both diphenolase (Fig. 4 ▸) and monophenolase (Fig. 5 ▸) activity. In both cases no browning of the crystals was observed, indicating that this mutation leads to loss of both activities over the measured time. This observation is in accordance with the theory that an aromatic system at the gatekeeper position of plant PPOs is important for the stabilization and the orientation of the substrate. The importance of this conserved phenylalanine for the activity of plant PPOs is in contrast to PPOs from other kingdoms (fungi and bacteria), where this position is not occupied by a phenyl­alanine.

The crystal structure of *Md*PPO1 will be the first of a plant tyrosinase in its latent form. The C-terminal structure will be of great interest as it could provide highly valuable information about the further functions of the C-terminal domain (besides its active-site shielding role) and the putative maturation site of the enzyme. This could enhance knowledge about the entire maturation process of plant tyrosinases. Furthermore, structure determination, comprehensive enzyme kinetic assays and the preparation of enzyme–inhibitor complex crystals for the wild type and the mutants are currently in progress and will hopefully provide some new structural insights into the monophenolase and diphenolase discrepancy between TYRs and COs and thus contribute to this longstanding scientific issue. The structures might reveal significant differences between the wild type and the mutants which could be used to deduce the reason for their differences in activity, which will be determined exactly using Michaelis–Menten kinetics. The differences in both the structures and the enzymatic activities upon mutation will improve knowledge of the catalytic event in plant PPOs; also, the crystal structures of inhibitor complexes of the enzyme may provide information to resolve many of the queries about substrate binding in the active centre of PPOs.

## Figures and Tables

**Table 1:** Production information for wild-type *Md*PPO1 and four mutants The amino acids in red indicate the positions of the respective mutations.

Protein	Wild-type *Md*PPO1	*Md*PPO1-Ala239Thr	*Md*PPO1-Leu243Arg	*Md*PPO1-Glu234Ala	*Md*PPO1-Phe259Ala
Source organism	*M. domestica* cv. Golden Delicious	*M. domestica* cv. Golden Delicious	*M. domestica* cv. Golden Delicious	*M.domestica* cv. Golden Delicious	*M. domestica* cv. Golden Delicious
DNA source	cDNA	Plasmid *Md*PPO1 in pGEX-6P-1	Plasmid *Md*PPO1 in pGEX-6P-1	Plasmid *Md*PPO1 in pGEX-6P-1	Plasmid *Md*PPO1 in pGEX-6P-1
Forward primer	5′-AGCCTATAGCCCCACCAGACG-3′	5′-GACACCACACACGCCGGTTCATTTATG-3′	5′-GCCGGTTCATAGATGGACCGGTG-3′	5′-TCCATCGCGGGGACACCA-3′	5′-GGGAATGCTTACTCCGCTGGT-3′
Reverse primer	5′-CTAAGAAGCAAATTCAATCTTGATACCACCAA-3′	5′-CCCTCGATGGAGCCGC-3′	5′-GCGTGTGGTGTCCCCTCG-3′	5′-GCCGCCACCAGGGTC-3′	5′-CATGTCCTCAAAGTTGGGCTG-3′
Cloning vector	pGEX-6P-1	pGEX-6P-1	pGEX-6P-1	pGEX-6P-1	pGEX-6P-1
Expression vector	pGEX-6P-1	pGEX-6P-1	pGEX-6P-1	pGEX-6P-1	pGEX-6P-1
Expression host	*E. coli* BL21	*E. coli* BL21	*E. coli* BL21	*E. coli* BL21	*E. coli* BL21
Complete amino acid sequence of the construct produced	MSPILGYWKIKGLVQPTRLLLEYLEEKYEEHLYERDEGDKWRNKKFELGLEFPNLPYYIDGDVKLTQSMAIIRYIADKHNMLGGCPKERAEISMLEGAVLDIRYGVSRIAYSKDFETLKVDFLSKLPEMLKMFEDRLCHKTYLNGDHVTHPDFMLYDALDVVLYMDPMCLDAFPKLVCFKKRIEAIPQIDKYLKSSKYIAWPLQGWQATFGGGDHPPKSDLEVLFQGPKPIAPPDVSKCGPADLPQGAVPTNCCPPPSTKIIDFKLPAPAKLRIRPPAHAVDQAYRDKYYKAMELMKALPDDDPRSFKQQAAVHCAYCDGAYDQVGFPELELQIHNSWLFFPFHRYYLYFFEKILGKLINDPTFALPFWNWDSPAGMPLPAIYADPKSPLYDKLRSANHQPPTLVDLDYNGTEDNVSKETTINANLKIMYRQMVSNSKNAKLFFGNPYRAGDEPDPGGGSIEGTPHAPVHLWTGDNTQPNFEDMGNFYSAGRDPIFFAHHSNVDRMWSIWKTLGGKRTDLTDSDWLDSGFLFYNENAELVRVKVRDCLETKNLGYVYQDVDIPWLSSKPTPRRAKVALSKVAKKLGVAHAAVASSSKVVAGTEFPISLGSKISTVVKRPKQKKRSKKAKEDEEEILVIEGIEFDRDVAVKFDVYVNDVDDLPSGPDKTEFAGSFVSVPHSHKHKKKMNTILRLGLTDLLEEIEAEDDDSVVVTLVPKFGAVKIGGIKIEFAS	MSPILGYWKIKGLVQPTRLLLEYLEEKYEEHLYERDEGDKWRNKKFELGLEFPNLPYYIDGDVKLTQSMAIIRYIADKHNMLGGCPKERAEISMLEGAVLDIRYGVSRIAYSKDFETLKVDFLSKLPEMLKMFEDRLCHKTYLNGDHVTHPDFMLYDALDVVLYMDPMCLDAFPKLVCFKKRIEAIPQIDKYLKSSKYIAWPLQGWQATFGGGDHPPKSDLEVLFQGPKPIAPPDVSKCGPADLPQGAVPTNCCPPPSTKIIDFKLPAPAKLRIRPPAHAVDQAYRDKYYKAMELMKALPDDDPRSFKQQAAVHCAYCDGAYDQVGFPELELQIHNSWLFFPFHRYYLYFFEKILGKLINDPTFALPFWNWDSPAGMPLPAIYADPKSPLYDKLRSANHQPPTLVDLDYNGTEDNVSKETTINANLKIMYRQMVSNSKNAKLFFGNPYRAGDEPDPGGGSIEGTPHTPVHLWTGDNTQPNFEDMGNFYSAGRDPIFFAHHSNVDRMWSIWKTLGGKRTDLTDSDWLDSGFLFYNENAELVRVKVRDCLETKNLGYVYQDVDIPWLSSKPTPRRAKVALSKVAKKLGVAHAAVASSSKVVAGTEFPISLGSKISTVVKRPKQKKRSKKAKEDEEEILVIEGIEFDRDVAVKFDVYVNDVDDLPSGPDKTEFAGSFVSVPHSHKHKKKMNTILRLGLTDLLEEIEAEDDDSVVVTLVPKFGAVKIGGIKIEFAS	MSPILGYWKIKGLVQPTRLLLEYLEEKYEEHLYERDEGDKWRNKKFELGLEFPNLPYYIDGDVKLTQSMAIIRYIADKHNMLGGCPKERAEISMLEGAVLDIRYGVSRIAYSKDFETLKVDFLSKLPEMLKMFEDRLCHKTYLNGDHVTHPDFMLYDALDVVLYMDPMCLDAFPKLVCFKKRIEAIPQIDKYLKSSKYIAWPLQGWQATFGGGDHPPKSDLEVLFQGPKPIAPPDVSKCGPADLPQGAVPTNCCPPPSTKIIDFKLPAPAKLRIRPPAHAVDQAYRDKYYKAMELMKALPDDDPRSFKQQAAVHCAYCDGAYDQVGFPELELQIHNSWLFFPFHRYYLYFFEKILGKLINDPTFALPFWNWDSPAGMPLPAIYADPKSPLYDKLRSANHQPPTLVDLDYNGTEDNVSKETTINANLKIMYRQMVSNSKNAKLFFGNPYRAGDEPDPGGGSIEGTPHAPVHRWTGDNTQPNFEDMGNFYSAGRDPIFFAHHSNVDRMWSIWKTLGGKRTDLTDSDWLDSGFLFYNENAELVRVKVRDCLETKNLGYVYQDVDIPWLSSKPTPRRAKVALSKVAKKLGVAHAAVASSSKVVAGTEFPISLGSKISTVVKRPKQKKRSKKAKEDEEEILVIEGIEFDRDVAVKFDVYVNDVDDLPSGPDKTEFAGSFVSVPHSHKHKKKMNTILRLGLTDLLEEIEAEDDDSVVVTLVPKFGAVKIGGIKIEFAS	MSPILGYWKIKGLVQPTRLLLEYLEEKYEEHLYERDEGDKWRNKKFELGLEFPNLPYYIDGDVKLTQSMAIIRYIADKHNMLGGCPKERAEISMLEGAVLDIRYGVSRIAYSKDFETLKVDFLSKLPEMLKMFEDRLCHKTYLNGDHVTHPDFMLYDALDVVLYMDPMCLDAFPKLVCFKKRIEAIPQIDKYLKSSKYIAWPLQGWQATFGGGDHPPKSDLEVLFQGPKPIAPPDVSKCGPADLPQGAVPTNCCPPPSTKIIDFKLPAPAKLRIRPPAHAVDQAYRDKYYKAMELMKALPDDDPRSFKQQAAVHCAYCDGAYDQVGFPELELQIHNSWLFFPFHRYYLYFFEKILGKLINDPTFALPFWNWDSPAGMPLPAIYADPKSPLYDKLRSANHQPPTLVDLDYNGTEDNVSKETTINANLKIMYRQMVSNSKNAKLFFGNPYRAGDEPDPGGGSIAGTPHAPVHLWTGDNTQPNFEDMGNFYSAGRDPIFFAHHSNVDRMWSIWKTLGGKRTDLTDSDWLDSGFLFYNENAELVRVKVRDCLETKNLGYVYQDVDIPWLSSKPTPRRAKVALSKVAKKLGVAHAAVASSSKVVAGTEFPISLGSKISTVVKRPKQKKRSKKAKEDEEEILVIEGIEFDRDVAVKFDVYVNDVDDLPSGPDKTEFAGSFVSVPHSHKHKKKMNTILRLGLTDLLEEIEAEDDDSVVVTLVPKFGAVKIGGIKIEFAS	MSPILGYWKIKGLVQPTRLLLEYLEEKYEEHLYERDEGDKWRNKKFELGLEFPNLPYYIDGDVKLTQSMAIIRYIADKHNMLGGCPKERAEISMLEGAVLDIRYGVSRIAYSKDFETLKVDFLSKLPEMLKMFEDRLCHKTYLNGDHVTHPDFMLYDALDVVLYMDPMCLDAFPKLVCFKKRIEAIPQIDKYLKSSKYIAWPLQGWQATFGGGDHPPKSDLEVLFQGPKPIAPPDVSKCGPADLPQGAVPTNCCPPPSTKIIDFKLPAPAKLRIRPPAHAVDQAYRDKYYKAMELMKALPDDDPRSFKQQAAVHCAYCDGAYDQVGFPELELQIHNSWLFFPFHRYYLYFFEKILGKLINDPTFALPFWNWDSPAGMPLPAIYADPKSPLYDKLRSANHQPPTLVDLDYNGTEDNVSKETTINANLKIMYRQMVSNSKNAKLFFGNPYRAGDEPDPGGGSIEGTPHAPVHLWTGDNTQPNFEDMGNAYSAGRDPIFFAHHSNVDRMWSIWKTLGGKRTDLTDSDWLDSGFLFYNENAELVRVKVRDCLETKNLGYVYQDVDIPWLSSKPTPRRAKVALSKVAKKLGVAHAAVASSSKVVAGTEFPISLGSKISTVVKRPKQKKRSKKAKEDEEEILVIEGIEFDRDVAVKFDVYVNDVDDLPSGPDKTEFAGSFVSVPHSHKHKKKMNTILRLGLTDLLEEIEAEDDDSVVVTLVPKFGAVKIGGIKIEFAS

**Table 2:** Crystallization conditions for latent wild-type *Md*PPO1 and the mutants *Md*PPO1-Ala239Thr, *Md*PPO1-Leu243Ala, *Md*PPO1-Glu234Ala and *Md*PPO1-Phe259Ala

Method	Vapour diffusion (hanging drop)
Plate type	15-well EasyXtal plates (Qiagen)
Temperature (K)	293
Protein concentration (mg ml^−1^)	5–10
Buffer composition of protein solution	50 m*M* Tris–HCl pH 7.5, 200 m*M* NaCl
Composition of reservoir solution	50 m*M* Tris–HCl pH 7.0, 19–21% PEG 3350
Volume and ratio of drop	1 µl protein solution, 2 µl reservoir solution
Volume of reservoir (µl)	500

**Table 3:** Data-collection and processing statistics for wild-type *Md*PPO1, *Md*PPO1-Ala239Thr and *Md*PPO1-Phe259Ala Values in parentheses are for the outer shell.

Protein	Wild-type *Md*PPO1	*Md*PPO1-Ala239Thr	*Md*PPO1-Phe259Ala
Diffraction source	ID23, ESRF	ID30A-3, ESRF	ID30A-3, ESRF
Wavelength (Å)	0.97242	0.96770	0.96770
Temperature (K)	100	100	100
Detector	PILATUS 6M	EIGER 4M	EIGER 4M
Crystal-to-detector distance (mm)	189.76	118.16	118.16
Rotation range per image (°)	0.1	0.1	0.1
Total rotation range (°)	320	300	300
Exposure time per image (s)	0.125	0.05	0.05
Space group	*P*2_1_2_1_2_1_	*P*2_1_2_1_2_1_	*P*2_1_2_1_2_1_
*a*, *b*, *c* (Å)	50.70, 80.15, 115.96	50.58, 79.90, 115.76	50.53, 79.76, 116.07
Mosaicity (°)	0.173	0.073	0.199
Resolution range (Å)	46.45–1.346	49.95–1.550	34.81–1.698
Total No. of reflections	1214526 (117878)	725427 (73222)	566074 (53670)
No. of unique reflections	104709 (10123)	67568 (6580)	51889 (5071)
Completeness (%)	99.51 (97.62)	98.2 (96.8)	98.6 (97.70)
Multiplicity	11.6 (11.6)	10.7 (11.1)	10.9 (10.6)
〈*I*/σ(*I*)〉	11.72 (2.02)	11.90 (1.50)	6.67 (1.06)
*R*_r.i.m._	0.120 (1.174)	0.119 (1.687)	0.257 (1.698)
*R*_p.i.m_ †	0.034 (0.337)	0.036 (0.495)	0.076 (0.505)
CC_1/2_ ‡	0.996 (0.708)	0.999 (0.564)	0.992 (0.478)
Overall *B* factor from Wilson plot (Å^2^)	14.00	20.27	18.89

† *R*_p.i.m._ = 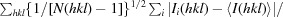 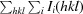, where *I_i_*(*hkl*) is the *i*th observation of reflection *hkl* and 〈*I*(*hkl*)〉 is the weighted average intensity for all observations of reflection *hkl*.

‡ CC_1/2_ is defined as the correlation coefficient between two random half data sets, as described by Karplus & Diederichs (2012 ▸).
